# Supplementary material for: Sugar-Sweetened Beverage Consumption in Adults: Evidence from a National Health Survey in Peru
Source: Nutrients. 2022 Jan 28;14(3):582. doi: 10.3390/nu14030582 (PMC8840136; doi:10.3390/nu14030582)
Supplement: Supplementary file 1 [file nutrients-14-00582-s001.zip › nutrients-1564136-supplementary.pdf]

Table S1. Summary statistics of the ready-to-drink SSB consumption/day in the first and second 24-h recall in those with two valid 24-h recalls.

	First 24-h recall	Second 24-h recall
**Minimum value**	0	0
**1st quartile**	0	0
**Median**	0	0
**Mean**	0.57	0.62
**3rd quartile**	0.67	0.92
**Maximum value**	5.84	7.6

*These statistics do not consider the survey sample design of the survey as those with two valid recalls were only 15% of the study population (139 out of 913 people).

Table S2. Summary statistics of the homemade SSB consumption/day in the first and second 24-hour recall in those with two valid 24-h recalls.

	First 24-h recall	Second 24-h recall
**Minimum value**	0	0
**1st quartile**	0	0
**Median**	1.06	1.18
**Mean**	1.32	1.25
**3rd quartile**	2.09	1.9
**Maximum value**	6.94	9.06

*These statistics do not consider the survey sample design of the survey as those with two valid recalls were only 15% of the study population (139 out of 913 people).

Table S3. Summary statistics of the first and second systolic (SBP) and diastolic blood pressure (DBP) records in the overall sample.

	First SBP record	Second SBP record	First DBP record	Second DBP record
**Minimum value**	71	72	50	50
**1st quartile**	98	97	65	64
**Median**	107	106	71	70
**Mean**	108.4	107.31	71.54	70.97
**3rd quartile**	117	116	77	76.5
**Maximum value**	203	198	118	124

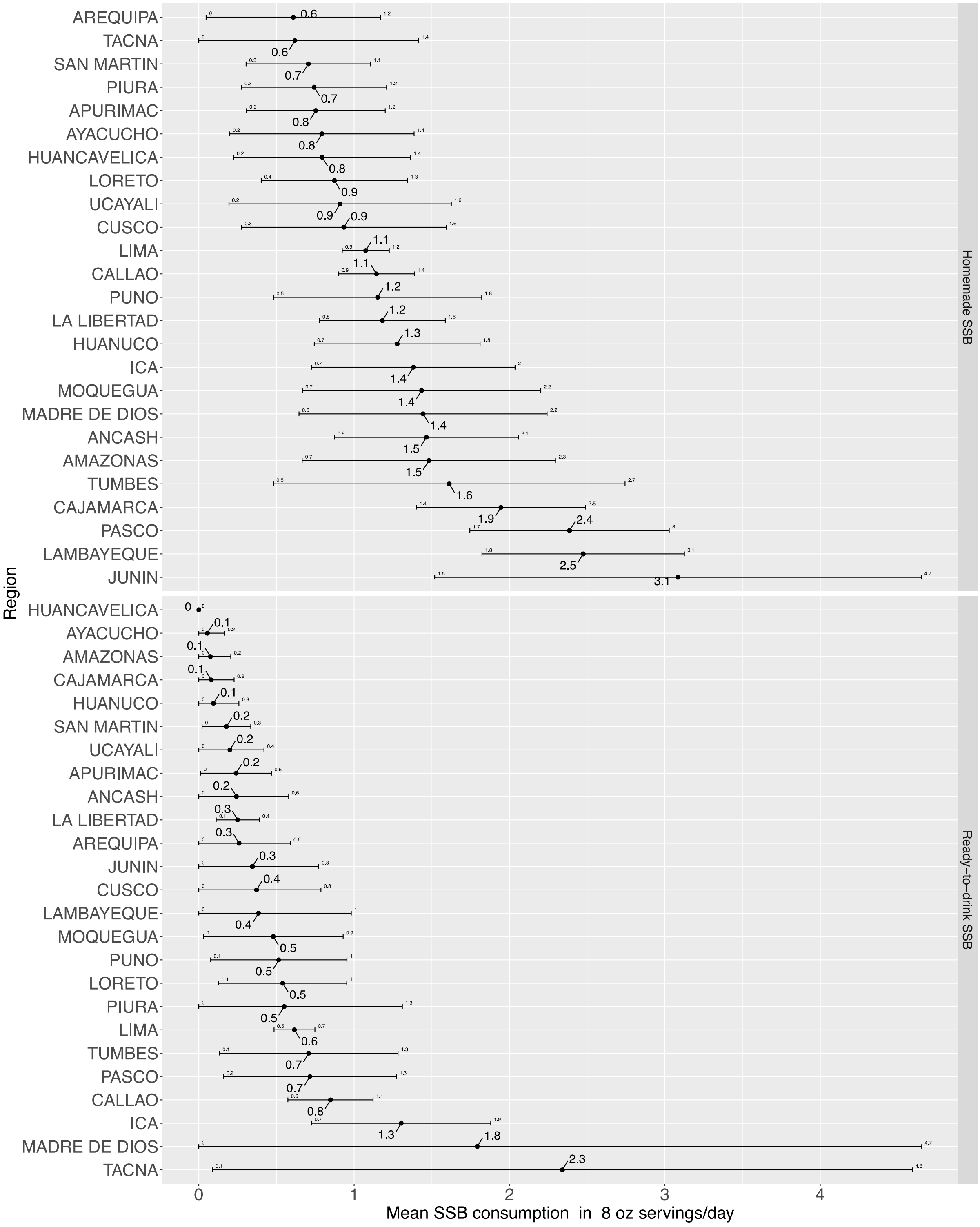

Figure S1. Mean consumption of homemade and ready-to-drink SSB according to Peruvian regions.

Table S4. Adjusted linear regression models regarding ready-to-drink SSB consumption.

	Estimate (8 oz. servings)	Standard error	*p* value
**Women (vs. men)**	−0.34	0.1	<0.001
**Age (in years)**	−0.01	0	0.08297
**Urban (vs. rural)**	0.35	0.08	<0.001
**Low education (vs. medium)**	−0.26	0.09	0.00383
**High education (vs. medium)**	−0.2	0.11	0.06200
**Overweight (vs. normal)**	0	0.1	0.98658
**Obesity (vs. normal)**	0.17	0.14	0.22500
**Abdominal obesity (vs. normal)**	0.06	0.11	0.59375
**Total diabetes (vs. no diabetes)**	0.16	0.15	0.27032
**Total hypertension (vs. no hypertension)**	−0.04	0.14	0.79553

Table S5. Crude linear regression models regarding ready-to-drink SSB consumption.

	Estimate (8 oz. servings)	Standard error	*p* value
**Women (vs. men)**	−0.37	0.09	<0.001
**Age (in years)**	−0.01	0	0.0297
**Urban (vs. rural)**	0.42	0.06	<0.001
**Low education (vs. medium)**	−0.51	0.08	<0.001
**High education (vs. medium)**	−0.17	0.1	0.0737
**Overweight (vs. normal)**	0.06	0.08	0.4319
**Obesity (vs. normal)**	0.25	0.11	0.0218
**Abdominal obesity (vs. normal)**	0.02	0.09	0.8488
**Total diabetes (vs. no diabetes)**	0.2	0.15	0.1883
**Total hypertension (vs. no hypertension)**	−0.02	0.11	0.8465

Table S6. Adjusted linear regression models regarding homemade SSB consumption.

	Estimate (8 oz. servings)	Standard error	*p* value
**Women (vs. men)**	−0.05	0.12	0.64216
**Age (in years)**	0.03	0.01	<0.001
**Urban (vs. rural)**	−0.18	0.15	0.24636
**Low education (vs. medium)**	−0.38	0.16	0.01877
**High education (vs. medium)**	0.21	0.12	0.08611
**Overweight (vs. normal)**	0.2	0.17	0.24800
**Obesity (vs. normal)**	−0.11	0.18	0.54284
**Abdominal obesity (vs. normal)**	−0.5	0.18	0.00638
**Total diabetes (vs. no diabetes)**	−0.14	0.18	0.43475
**Total hypertension (vs. no hypertension)**	0.02	0.16	0.88888

Table S7. Crude linear regression models regarding homemade SSB consumption.

	Estimate (8 oz. servings)	Standard error	*p* value
**Women (vs. men)**	−0.23	0.11	0.0287
**Age (in years)**	0.02	0.01	<0.001
**Urban (vs. rural)**	−0.11	0.13	0.4006
**Low education (vs. medium)**	−0.17	0.14	0.2162
**High education (vs. medium)**	0.1	0.12	0.4292
**Overweight (vs. normal)**	−0.05	0.14	0.7383
**Obesity (vs. normal)**	−0.29	0.14	0.0387
**Abdominal obesity (vs. normal)**	−0.21	0.13	0.1038
**Total diabetes (vs. no diabetes)**	−0.08	0.18	0.6476
**Total hypertension (vs. no hypertension)**	−0.04	0.16	0.8270

Table S8. Ready-to-drink SSB consumption in men and women by socio-demographic and health-related variables.

Variable	Options for variable	Sex	Number of observations	SSB consumption (mean and 95% CI)	*p* value*
Sex	Men	-	386	0.71 (0.57–0.86)	<0.05
	Women	-	527	0.35 (0.26–0.43)	
Age group	18–29	Men	116	0.74 (0.52–0.96)	<0.05
	30–39	Men	97	0.96 (0.56–1.37)	
	40–49	Men	87	0.58 (0.3–0.87)	
	50–59	Men	86	0.51 (0.24–0.78)	
	18–29	Women	142	0.38 (0.22–0.53)	0.42
	30–39	Women	142	0.27 (0.14–0.39)	
	40–49	Women	139	0.42 (0.24–0.59)	
	50–59	Women	104	0.32 (0.16–0.49)	
	18–29	Total	258	0.55 (0.42–0.68)	0.15
	30–39	Total	239	0.55 (0.37–0.73)	
	40–49	Total	226	0.47 (0.33–0.62)	
	50–59	Total	190	0.41 (0.26–0.55)	
Urban/rural	Rural	Men	142	0.22 (0.12–0.33)	<0.05
	Urban	Men	244	0.86 (0.67–1.04)	
	Rural	Women	184	0.13 (0.06–0.21)	<0.05
	Urban	Women	343	0.4 (0.3–0.51)	
	Rural	Total	326	0.17 (0.1–0.24)	<0.05
	Urban	Total	587	0.59 (0.49–0.69)	
Educational level	Low	Men	67	0.2 (0.03–0.37)	<0.05
	Medium	Men	167	0.95 (0.7–1.21)	
	High	Men	152	0.66 (0.43–0.88)	
	Low	Women	160	0.15 (0.05–0.25)	<0.05
	Medium	Women	172	0.43 (0.28–0.57)	
	High	Women	194	0.39 (0.26–0.52)	
	Low	Total	227	0.17 (0.08–0.25)	<0.05
	Medium	Total	339	0.68 (0.53–0.82)	
	High	Total	346	0.5 (0.38–0.63)	
Body mass index category	Normal	Men	163	0.6 (0.37–0.82)	<0.05
	Overweight	Men	144	0.63 (0.45–0.81)	
	Obesity	Men	76	1.06 (0.62–1.49)	
	Normal	Women	166	0.24 (0.12–0.36)	<0.05
	Overweight	Women	197	0.35 (0.21–0.49)	
	Obesity	Women	158	0.45 (0.29–0.6)	
	Normal	Total	329	0.41 (0.29–0.53)	<0.05
	Overweight	Total	341	0.47 (0.36–0.59)	
	Obesity	Total	234	0.66 (0.48–0.84)	

Abdominal obesity	No	Men	185	0.62 (0.41–0.82)	<0.05
	Yes	Men	201	0.79 (0.58–0.99)	
	No	Women	93	0.28 (0.15–0.4)	0.95
	Yes	Women	409	0.37 (0.27–0.48)	
	No	Total	278	0.5 (0.36–0.64)	0.84
	Yes	Total	610	0.52 (0.42–0.62)	
Diabetes status	Not with diabetes	Men	157	0.64 (0.49–0.79)	<0.05†
	Total diabetes	Men	229	1.1 (0.54–1.65)	
	Self-reported diabetes	Men	14	0.81 (0.24–1.39)	
	Unaware diabetes	Men	215	1.2 (0.48–1.93)	
	Total diabetes	Women	289	0.34 (0.13–0.56)	0.18†
	Unaware diabetes	Women	262	0.48 (0.12–0.83)	
	Not with diabetes	Women	238	0.38 (0.28–0.48)	
	Self-reported diabetes	Women	26	0.18 (0–0.4)	
	Self-reported diabetes	Total	40	0.4 (0.12–0.67)	<0.05†
	Not with diabetes	Total	395	0.49 (0.41–0.57)	
	Total diabetes	Total	518	0.69 (0.4–0.98)	
	Unaware diabetes	Total	477	0.86 (0.43–1.28)	
Hypertension status	Not with hypertension	Men	335	0.74 (0.58–0.91)	0.62‡
	Total hypertension	Men	45	0.49 (0.23–0.75)	
	Self-reported hypertension	Men	30	0.43 (0.14–0.71)	
	Unaware hypertension	Men	15	0.63 (0.11–1.15)	
	Not with hypertension	Women	453	0.35 (0.25–0.44)	0.31‡
	Total hypertension	Women	51	0.49 (0.19–0.8)	
	Self-reported hypertension	Women	41	0.52 (0.16–0.88)	
	Unaware hypertension	Women	10	0.39 (0.02–0.75)	
	Not with hypertension	Total	788	0.51 (0.42–0.6)	0.63‡

Self-reported hypertension	Total	71	0.47 (0.25–0.7)
Total hypertension	Total	96	0.49 (0.3–0.68)
Unaware hypertension	Total	25	0.55 (0.19–0.91)

*For dichotomous variables (sex, urban/rural, abdominal obesity), *p* value from unpaired t-test. For the remaining variables except diabetes and hypertension status, *p* value for ANOVA test. †*p* value for t-test between total diabetes and not with diabetes ‡*p* value for t-test between total hypertension and not with hypertension.

Table S9. Homemade SSB consumption in men and women by socio-demographic and health-related variables.

Variable	Options for variable	Sex	Number of observations	SSB consumption (mean and 95% CI)	*p* value
Sex	Men	-	386	1.3 (1.12–1.47)	<0.05
	Women	-	527	1.06 (0.93–1.19)	
Age group	18–29	Men	116	0.97 (0.73–1.21)	<0.05
	30–39	Men	97	1.17 (0.83–1.52)	
	40–49	Men	87	1.35 (0.96–1.74)	
	50–59	Men	86	1.89 (1.4–2.38)	
	18–29	Women	142	0.89 (0.65–1.14)	0.37
	30–39	Women	142	1.02 (0.8–1.25)	
	40–49	Women	139	1.09 (0.81–1.38)	
	50–59	Women	104	1.31 (1–1.61)	
	18–29	Total	258	0.93 (0.76–1.1)	<0.05
	30–39	Total	239	1.08 (0.89–1.28)	
	40–49	Total	226	1.18 (0.95–1.42)	
	50–59	Total	190	1.57 (1.27–1.86)	
Urban/rural	Rural	Men	142	1.62 (1.29–1.96)	0.08
	Urban	Men	244	1.2 (1–1.4)	
	Rural	Women	184	0.94 (0.72–1.17)	0.51
	Urban	Women	343	1.09 (0.93–1.25)	
	Rural	Total	326	1.24 (1.03–1.46)	0.32
	Urban	Total	587	1.14 (1.01–1.27)	
Educational level	Low	Men	67	1.16 (0.76–1.57)	0.45
	Medium	Men	167	1.38 (1.1–1.66)	
	High	Men	152	1.26 (0.99–1.53)	
	Low	Women	160	0.89 (0.65–1.14)	0.07
	Medium	Women	172	0.94 (0.76–1.12)	
	High	Women	194	1.24 (1.01–1.46)	
	Low	Total	227	0.97 (0.75–1.2)	0.14
	Medium	Total	339	1.15 (0.98–1.32)	
	High	Total	346	1.25 (1.07–1.43)	
Body mass index category	Normal	Men	163	1.32 (1.03–1.61)	0.39
	Overweight	Men	144	1.4 (1.08–1.71)	

	Obesity	Men	76	1.09 (0.73–1.45)	
	Normal	Women	166	1.21 (0.95–1.47)	
	Overweight	Women	197	1.08 (0.85–1.3)	0.65
	Obesity	Women	158	0.9 (0.7–1.1)	
	Normal	Total	329	1.26 (1.05–1.47)	
	Overweight	Total	341	1.21 (1.02–1.4)	0.17
	Obesity	Total	234	0.97 (0.79–1.15)	
Abdominal obesity	No	Men	185	1.42 (1.15–1.69)	0.06
	Yes	Men	201	1.2 (0.97–1.43)	
	No	Women	93	1.14 (0.79–1.5)	0.95
	Yes	Women	409	1.07 (0.92–1.22)	
	No	Total	278	1.32 (1.11–1.54)	<0.05
	Yes	Total	610	1.12 (0.98–1.25)	
Diabetes status	Not with diabetes	Men	157	1.3 (1.1–1.5)	0.80†
	Total diabetes	Men	229	1.18 (0.73–1.64)	
	Self-reported diabetes	Men	14	0.81 (0.15–1.46)	
	Unaware diabetes	Men	215	1.32 (0.73–1.91)	
	Not with diabetes	Women	238	1.08 (0.94–1.23)	0.25†
	Total diabetes	Women	289	1.02 (0.56–1.48)	
	Self-reported diabetes	Women	26	0.73 (0.26–1.2)	
	Unaware diabetes	Women	262	1.25 (0.56–1.94)	
	Not with diabetes	Total	395	1.18 (1.05–1.3)	0.67†
	Self-reported diabetes	Total	40	0.76 (0.39–1.13)	
	Total diabetes	Total	518	1.09 (0.77–1.42)	
	Unaware diabetes	Total	477	1.29 (0.84–1.74)	
Hypertension status	Not with hypertension	Men	335	1.29 (1.09–1.48)	0.59‡
	Total hypertension	Men	45	1.24 (0.78–1.71)	
	Self-reported hypertension	Men	30	1.3 (0.69–1.91)	
	Unaware hypertension	Men	15	1.11 (0.45–1.77)	
	Not with hypertension	Women	453	1.07 (0.93–1.21)	0.79‡

Total hypertension	Women	51	1 (0.56–1.43)	
Self-reported hypertension	Women	41	1 (0.48–1.52)	
Unaware hypertension	Women	10	0.99 (0.5–1.47)	
Total hypertension	Total	96	1.13 (0.83–1.42)	
Self-reported hypertension	Total	71	1.14 (0.79–1.5)	0.63‡
Not with hypertension	Total	788	1.16 (1.04–1.28)	
Unaware hypertension	Total	25	1.07 (0.6–1.54)	

*For dichotomous variables (sex, urban/rural, abdominal obesity), *p* value from unpaired t-test. For the remaining variables except diabetes and hypertension status, p value for ANOVA test. †*p* value for t-test between total diabetes and not with diabetes ‡*p* value for t-test between total hypertension and not with hypertension.

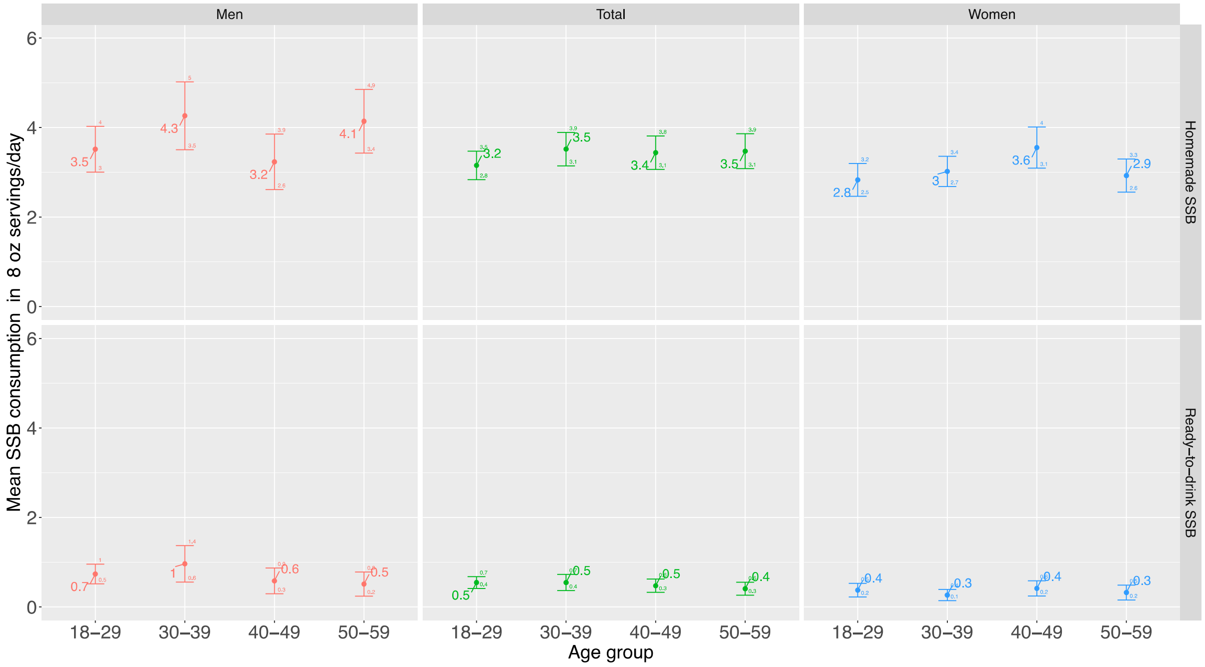

Figure S2. Sensitivity analysis. Mean consumption of homemade and ready-to-drink SSB according to age groups.

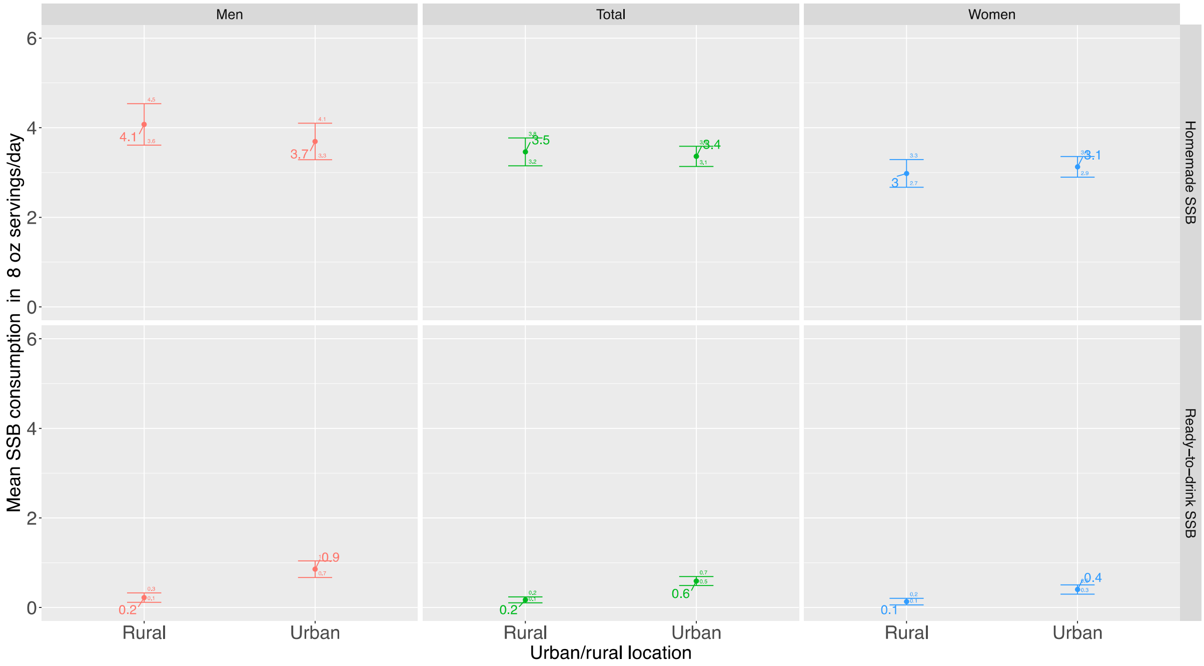

Figure S3. Sensitivity analysis. Mean consumption of homemade and ready-to-drink SSB according to urban/rural location.

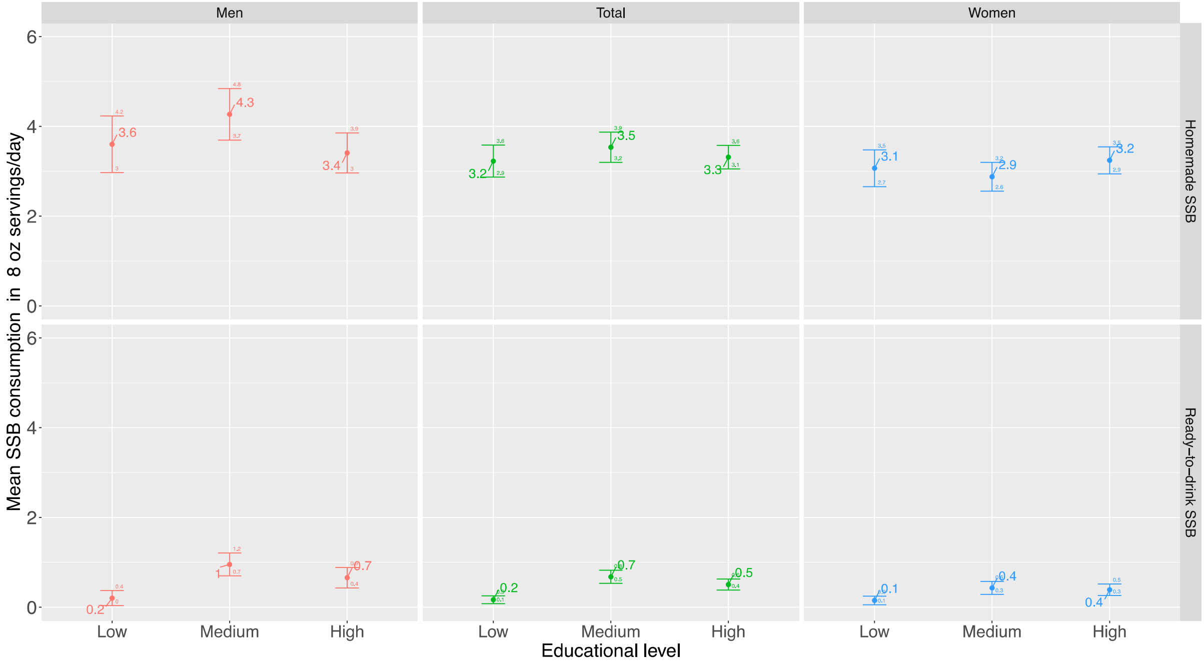

Figure S4. Sensitivity analysis. Mean consumption of homemade and ready-to-drink SSB according to educational level.

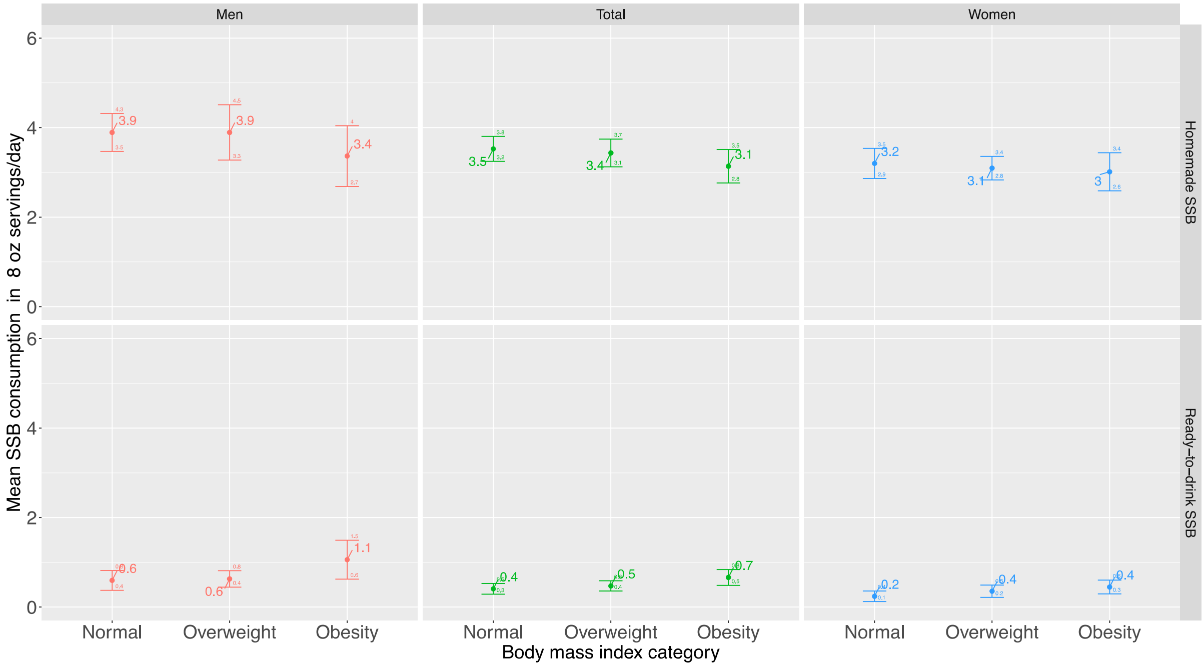

Figure S5. Sensitivity analysis. Mean consumption of homemade and ready-to-drink SSB according to body mass index categories.

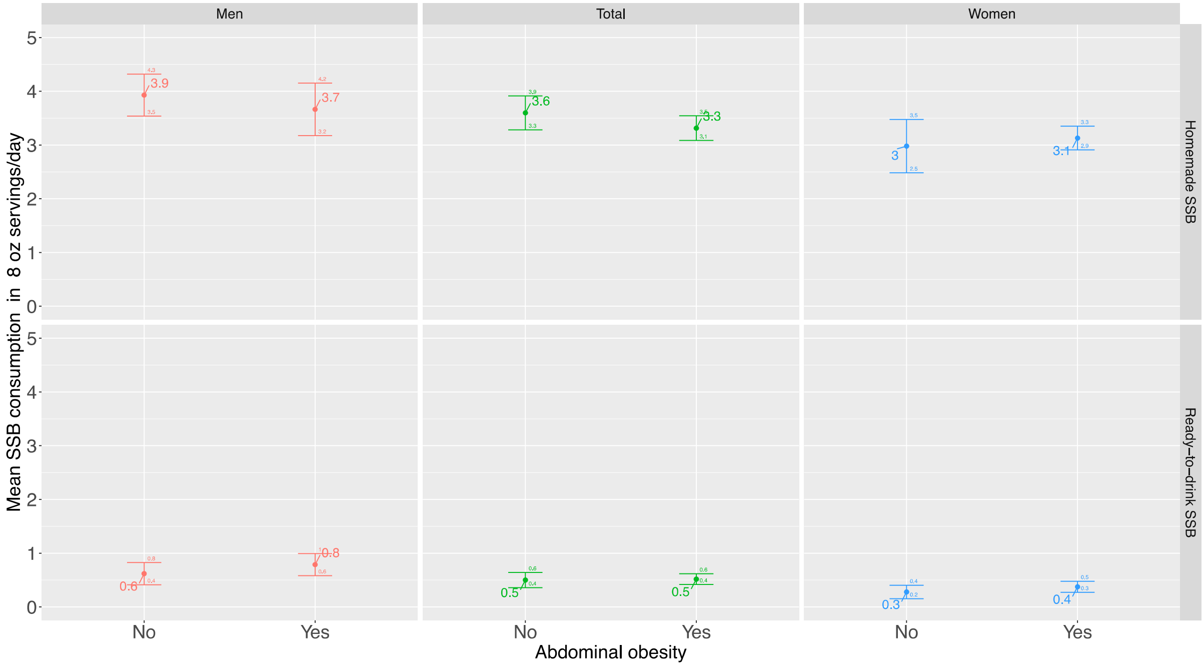

Figure S6. Sensitivity analysis. Mean consumption of homemade and ready-to-drink SSB according to abdominal obesity.

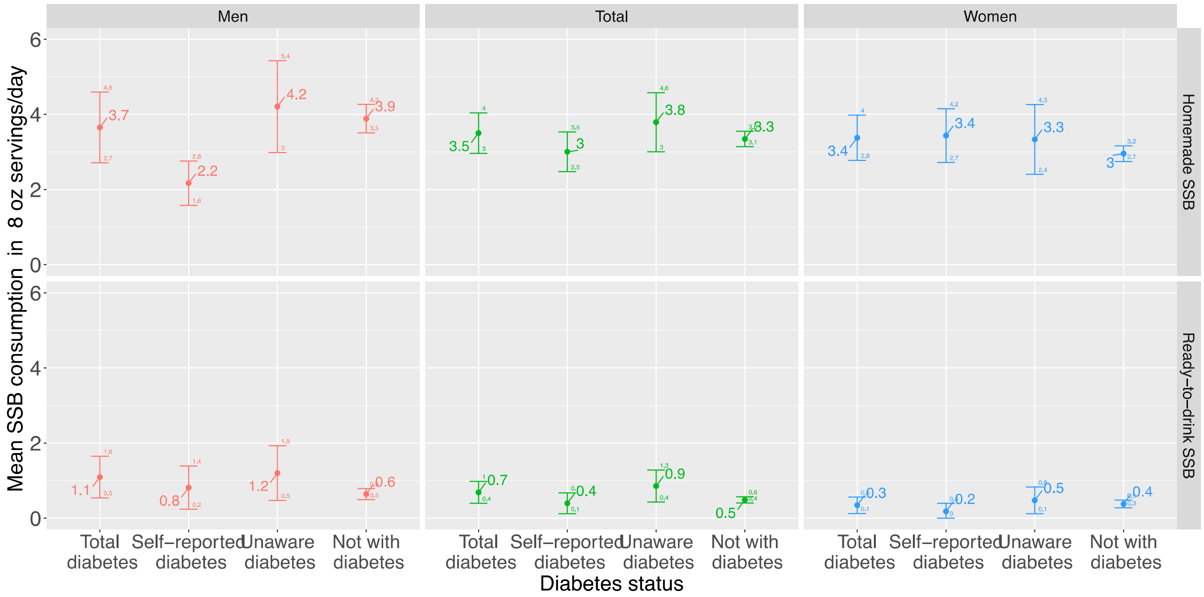

Figure S7. Sensitivity analysis. Mean consumption of homemade and ready-to-drink SSB according to diabetes status.

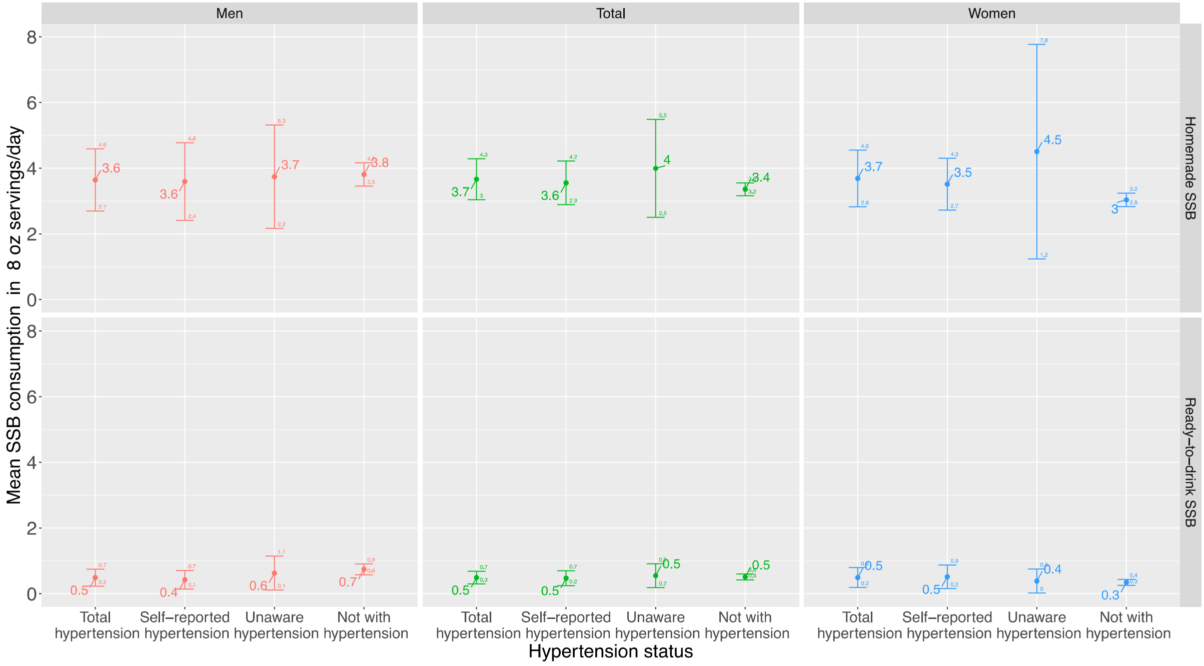

Figure S8. Sensitivity analysis. Mean consumption of homemade and ready-to-drink SSB according to hypertension status.

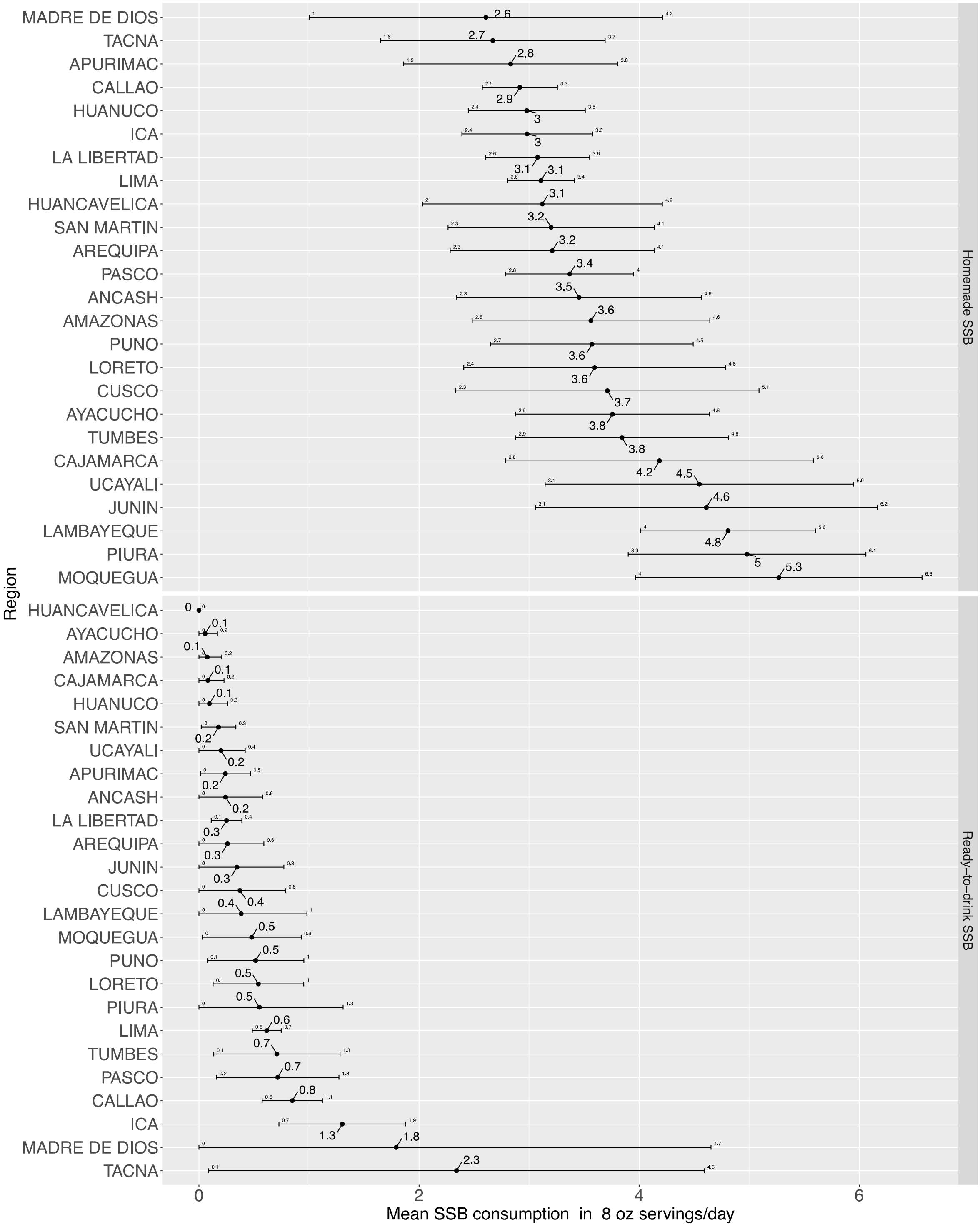

Figure S9. Sensitivity analysis. Mean consumption of homemade and ready-to-drink SSB according to Peruvian regions.
